# Huangqi-Honghua Combination Prevents Cerebral Infarction with Qi Deficiency and Blood Stasis Syndrome in Rats by the Autophagy Pathway

**DOI:** 10.1155/2022/9496926

**Published:** 2022-01-24

**Authors:** Yue Chen, Lu Lei, Kai Wang, Ruimin Liang, Yi Qiao, Zhijun Feng, Juanli Zhang, Min Bai, Haixia Chen, Jiaxin Zhao, Xingzhao Xiong, Jinyi Cao, Xia Shen, Zhifu Yang

**Affiliations:** ^1^College of Pharmacy, Shaanxi University of Chinese Medicine, Xianyang 712046, China; ^2^Department of Pharmacy, Xijing Hospital, Fourth Military Medical University, Xi'an 710032, China; ^3^Department of Pharmacy, Xi'an Central Hospital, Xi'an 710003, China; ^4^Department of Pharmacy, Xi'an Daxing Hospital, Xi'an 710016, China; ^5^Department of Chemistry and Biochemistry, University of Delaware, Newark, DE 19716, USA

## Abstract

**Background:**

Cerebral ischemia/reperfusion injury (CI/RI) contributes to the process of autophagy. Huangqi-Honghua combination (HQ-HH) is a traditional Chinese medicine (TCM) combination that has been widely used in the treatment of cerebrovascular diseases in China. The role of autophagy in HQ-HH-mediated treatment of CI/RI is unclear.

**Methods:**

Sprague-Dawley (SD) rats were used to establish the middle cerebral artery occlusion (MCAO) with QDBS syndrome model and evaluate the function of HQ-HH in protecting against CI/RI.

**Results:**

HQ-HH significantly improved the neuronal pathology and reduced infarct volume, neurological deficits, and whole blood viscosity in rats with CI/RI. Western blot results showed that the expression of autophagy marker proteins LC3II/LC3I and Beclin1 in the HQ-HH group was significantly lower than that in the model group, while the expression of p62 was significantly higher in the HQ-HH group as compared with the model group. There were no significant differences in PI3K, Akt, and mTOR levels between the HQ-HH group and the model group; however, p-PI3K, p-Akt, and p-mTOR were significantly upregulated. In addition, HQ-HH also changed the composition and function of intestinal flora in MCAO + QDBS model rats.

**Conclusion:**

HQ-HH protects from CI/RI, and its underlying mechanism may involve the activation of the PI3K-Akt-mTOR signaling pathway, relating to the changes in the composition of intestinal flora.

## 1. Introduction

Stroke is an acute cerebrovascular disease that can cause permanent neurological dysfunction or death. Stroke is a major threat to public health because of the associated high morbidity, disability, and recurrence rates [1]. Almost 80% of stroke is caused by ischemia due to vascular obstruction resulting in the disruption of blood flow to the brain [2]. Recanalization, a technique to restore brain blood flow and the major treatment approach, has a narrow time window. Neuroprotection therapies are the available alternative treatment options and the focus of current research, but nearly all clinical trials of neuroprotection therapies have failed. Therefore, none of the neuroprotection treatments is recommended in the latest guidelines for the early management of patients with acute ischemic stroke [3]. Therefore, exploring the underlying pathological mechanisms of stroke and finding effective treatments of stroke are the top healthcare research priorities [4].

Due to physiological complexity of ischemic stroke, research has gradually shifted from a focus on monotherapy to a combined or multitherapy approach since the synergy of therapeutic agents or techniques gives rise to ostentatious superadditive therapeutic effects (i.e., “1 + 1 > 2”) [5]. TCM is a holistic concept and a branch of traditional medicine in China. TCM has been widely used for the treatment of complex diseases, especially for the prevention and treatment of ischemic stroke. In the TCM theory, ischemic stroke is considered a type of QDBS syndrome [6], characterized by pale or dark skin, fatigue, lack of breath, palpitations, insomnia, and chest pain. The treatment methods mainly involve tonification of Qi, activation of blood circulation, and removal of blood stasis. *Radix Astragali* (Huangqi, HQ), the dry root of *Astragalus membranaceus* (Fisch.) Bge., is a traditional Chinese medicine used for tonifying Qi, which rebalances it and promotes blood circulation. This plant can be used to treat patients with stroke or chronic debilitating diseases. *Carthamus tinctorius* L. (Honghua, HH), a dried flower, promotes blood circulation and removes blood stasis. The combination of HQ and HH has been widely used in the treatment of cerebrovascular diseases with QDBS in China [7]. HQ and HH are the primary active constituents in Buyang Huanwu decoction [8], a well-known TCM formula, reported in “Yilin Gaicuo” written by Qingren Wang [9]. So, the HQ-HH combination fully fits the pathological mechanism of treating ischemic stroke with QDBS. In our previous study, we demonstrated the therapeutic activity of HQ-HH against stroke in vitro [10]. In our previous study, we used water extract of HQ and HH (5 : 1), which is very popular in clinical practice because of its ease of administration.

Autophagy activation is closely associated with the onset and development of CI/RI [11]. However, the role of autophagy in cerebral ischemia remains controversial [12]. Chronic or mild ischemia leads to autophagy [13] and protects the nerve cells by removing the damaged tissues and proteins. Moreover, severe ischemia may lead to excessive autophagy [14]. In addition, reactive oxygen species (ROS) play a signaling role in autophagy; treatment with antioxidants prevents the formation of autophagosomes and the subsequent degradation of proteins [15]. In our previous study, we found that HQ-HH exerts its neuroprotective effects because of its antioxidant properties. For instance, HQ-HH increases the activity of antioxidant enzymes-SOD, catalase, and GSH-Px and decreases the levels of MDA and ROS by upregulating the expression of Nrf2 [16].

Investigating the role of autophagy in HQ-HH-mediated cerebral ischemia treatment is the main aim of the present study. This study uses the MCAO with QDBS model, which simulates the stroke syndrome of patients to demonstrate the neuroprotective effect of HQ-HH, elucidate the underlying mechanism, and investigate the role of autophagy. Furthermore, current evidence suggests that gut microbiota can modulate stroke symptoms and behaviors through bidirectional gut-brain axis communication [17]. Gut microbiome dysbiosis plays a critical role in the development of many stroke-related events before the major stroke event [18]. So, we used 16S rRNA gene sequencing to analyze the effect of HQ-HH on intestinal microflora of rats with MCAO and understand the influence of “brain-gut-axis.”

## 2. Materials and Methods

### 2.1. Animals

We purchased male Sprague-Dawley (SD) rats (250–280g) from the Experimental Animal Center of People's Liberation Army Air Force Medical University (Xi'an, Shaanxi, China). All rats were housed at the Experimental Animal Center of the Pharmacy Department of the First Affiliated Hospital of the Air Force Military Medical University of the Chinese People's Liberation Army (Xi'an, Shaanxi, China). The breeding temperature was kept between 22 and 24 °C, and the relative humidity was set to 45 ± 5%. All experimental procedures were carried out according to the protocols approved by the Ethics Committee for Animal Experimentation of the Chinese People's Liberation Army Air Force Military Medical University (Xi'an, Shaanxi, China). The experiments were performed according to the National Institutes of Health Guide for the Care and Use of Laboratory Animals.

### 2.2. Development of MCAO with QDBS Syndrome Model

After a week of adaptive feeding, the rats were randomly categorized into five groups (*n* = 6): model group (MCAO + QDBS), sham group, HQ group, HH group, and HQ-HH group. Rats of all groups except the sham group were placed in water at (20 ± 1) °C and allowed to swim until exhaustion. These rats were then subjected to adaptive swimming training for 3 days and exhaustive swimming once a day for 21 days. On the 22nd day, the modified Longa suture method was used to block the right middle cerebral artery of the rats to establish the MCAO model [19]. The rats were fasted a day before but allowed to take water to ensure the successful completion of the anesthesia procedure. The animals were anesthetized by intraperitoneal injection of 3% sodium pentobarbital (Merck Company, USA) and placed in dorsal recumbency. The rectal temperature of the rats was maintained at 37.0 ± 0.5°C and monitored throughout the entire procedure. The rats were supinely fixed on the rat plate and disinfected with iodophor (Dezhou Gelijie Disinfection Products Co., Ltd., China). 1.5 cm cuts were made in the middle of the necks of the rats. Using the blunt instrument, the right common carotid artery (CCA), external carotid artery (ECA), and internal carotid artery (ICA) were separated. Small incisions were made in the right common carotid artery (CCA). The nylon wire (Ethicon, Inc., Osaka, Japan) was then inserted into the ICA through the CCA residue. The changes in cerebral blood flow were recorded by a laser Doppler flow meter (LDF, Moor Instruments, UK). If local cerebral blood flow dropped sharply to 30% of the baseline levels (preischemic), occlusion of the middle cerebral artery was considered appropriate. After 2 hours of ischemia, the nylon wire was carefully removed to achieve reperfusion. In the sham group, all steps except for the insertion of nylon wire were the same. After the procedure, rats were grouped into cages to ensure sufficient food and water.

### 2.3. Administration of Test Substances

HQ extract, HH extract, and HQ-HH extract were provided by Guangdong Yifang Pharmaceutical Co., Ltd. (Guangdong, China). Rats were intragastrically administered with 10 ml/kg of HQ-HH. The drug concentration of the administration groups was 0.437 g/ml for the HH group, 0.113 g/ml for the HH group, and 0.437 g/ml (HQ) + 0.113 g/ml (HH) for the HQ-HH group. Rats were subjected to exhaustive swimming for 21 days followed by the intragastric administration of HQ/HH/HQ-HH on day 13 for 9 consecutive days. Rats in the sham group and model group were given an equal volume of 0.9% saline (Shandong Chenxin Pharmaceutical Co., Ltd., China) daily. Focal cerebral ischemia was induced by MCAO on day 22. Samples were collected 24 h after administration on the 9th day, after which the rats were sacrificed.

### 2.4. Assessment of Neurological Deficit Score

Using the Zea-Longa method [20], we evaluated the neurological function of the rats that recovered from anesthesia after 24 hours of ischemia-reperfusion and determined the neurological deficit score. The degree of nerve damage in rats is positively correlated with the score, that is, the more severe the nerve damage, the higher the score. The scoring criteria of neurological impairment in rats are as follows: 0 points (level 0): no neurological deficit symptoms; 1 point (level 1): contralateral forelimb of the rat is flexed and the shoulder is internally rotated when the tail is lifted; 2 points (level 2): the shoulder of the operated side of the rat is pushed to move to the opposite side on a smooth surface, and the resistance decreases; 3 points (level 3): dumping or making a circle on the opposite side when walking freely; and 4 points (level 4): loss of consciousness, unable to carry out spontaneous activities.

### 2.5. Measurement of Infarction Volume

The brain tissues were extracted from the rats and frozen at −20°C for 10 min. The tissues were then placed in the brain groove, and each tissue was cut into five coronal slices using a sharp knife. The tissues were treated with 2% 2,3,5-triphenyl tetrazolium chloride (TTC) solution (Sigma, USA) and incubated at room temperature for 30 min under shade. The glass dishes were shaken once every 10 min to ensure full coloring of the tissues. The sections were fixed with 4% paraformaldehyde (Hefei White Shark Biotechnology Co., Ltd., China) and photographed using a digital camera. Image-Pro Plus software was used to measure the area of cerebral infarction.

Cerebral infarction volume (%) = (normal hemisphere volume − ischemia side nonischemia volume)/normal hemisphere volume × 100%.

### 2.6. Collection of Blood Samples

After 24 hours of I/R, blood was collected from the abdominal aorta of rats of each group using heparin lithium as an anticoagulant. The whole blood viscosities of rats at different shear rates (1S^−1^, 5S^−1^, 30S^−1^, and 200S^−1^) were calculated using an automatic hemorheology tester (Thermo Fisher Scientific Co., Ltd., USA).

### 2.7. Hematoxylin-Eosin (HE) Staining

The rat hearts were fixed by intraperitoneal injection twenty-four hours after CI/RI under anesthesia. Briefly, the heart was fully exposed by tearing off the pericardium and carefully fixed with dressing forceps. After inserting the perfusion needle into the apex of the heart, we cut open the right auricle of the rats and injected 0.9% normal saline (Shandong Chenxin Pharmaceutical Co., Ltd., China) at a uniform rate. We replaced the normal saline with 4% paraformaldehyde fixation solution to fix the brain tissues. After the brain tissues were fixed, decapitation and brain exercises were performed, and the removed brain tissues were fixed in 4% formaldehyde solution. After routine paraffin embedding and slicing, the brain tissue slides were inserted into the slide holder and dewaxed with xylene I (10 min, Tianjin Fuyu Fine Chemical Co., Ltd., China) and xylene II (5 min, Tianjin Fuyu Fine Chemical Co., Ltd., China). The samples were rehydrated with gradient alcohol (100%, 95%, 90%, 80%, 70%, and 50% alcohol, each time 3 min), soaked in hematoxylin aqueous solution (5 min, Shanghai Biyuntian Biotechnology Co., Ltd., China), and rinsed with tap water for 30 s twice. These samples were treated with gradient alcohol (50%, 70%, 80%, 90%, and 95%, each time 1 min), eosin staining solution (Shanghai Biyuntian Biotechnology Co., Ltd., China) for 30 s, and gradient alcohol (95%, 100% I, and 100% II, each 1 min) again to dehydrate. Finally, the samples were treated with transparent xylene (2 min, two times), sealed with neutral resin, dried, and observed under the light microscope.

### 2.8. Western Blotting

The cortical tissues of the rats were lysed with cell lysate buffer containing protease inhibitor. The lysate obtained was centrifuged at 12000 rpm for 20 min, and the supernatant was collected for Western blotting. Protein content in the samples was quantified using a BCA kit (Thermo Fisher, USA). Subsequently, proteins with different molecular weights were separated by sodium dodecyl sulfate-polyacrylamide gel electrophoresis (SDS-PAGE, Shanghai Biyuntian Biotechnology Co., Ltd., China) at different concentrations and transferred to polyvinylidene fluoride (PVDF, Millipore, USA) membrane. The membrane was blocked with 5% skim milk at 37 °C for 1 h and incubated with LC3II/LC3I (Abcam, ab192890), Beclin-1 (Abcam, ab207612), p62 (Abcam, ab109012), PI3K (Abcam, ab191606), Akt (Proteintech, 10174-2-AP), mTOR (Abcam, ab137133), and *β*-actin (Sigma-Aldrich, A5316) antibodies at 4°C overnight. The membrane was treated with goat anti-mouse or anti-rabbit peroxidase-conjugated secondary antibodies (Zhuangzhi Biological Technology Co., Ltd., Xi ‘an, China). Finally, immunoreactive protein bands were visualized using chemiluminescence reagent (ECL, Zeta Life, USA). ImageJ software was used to quantify the bands, and the staining intensity of the bands was evaluated by density measurement.

### 2.9. Gut Microbiota Analysis Using 16S rRNA Sequencing

All the fecal samples were subjected to the same procedures for DNA extraction and PCR amplification. The total DNA was extracted from the contents of the rat cecum using E.Z.N.A.® Stool DNA Kit (Omega Bio-Tek, Inc., GA, USA) as per instructions. The extracted DNA from each sample was used as the template to amplify the V3∼V4 region of 16S rRNA genes. The primers F1 and R2 (5′-CCTACGGGNGGCWGCAG-3′ and 5′-GACTACHVGGGTATCTAATCC-3′) corresponding to positions 341 to 805 in the *Escherichia coli* 16S rRNA gene were used to amplify the V3∼V4 region of each fecal sample by PCR. PCR reactions were run in the EasyCycler 96 PCR system (Analytik Jena AG Corp.) using the following program: 3 min of denaturation at 95°C followed by 21 cycles of 0.5 min at 94°C (denaturation), 0.5 min of annealing at 58°C, and 0.5 min at 72°C (elongation), with a final extension at 72°C for 5 min. The products from different samples were indexed and mixed at equal ratios and sequenced by Shanghai Mobio Biomedical Technology Co., Ltd., using the MiSeq platform (Illumina Inc., USA). Clean data was extracted from the raw data using USEARCH 8.0. Quality-filtered sequences were clustered into unique sequences and sorted in order of decreasing abundance to identify representative sequences using UPARSE according to the UPARSE operational taxonomic unit (OTU) analysis pipeline. The singletons were also omitted in this step. OTUs were classified based on 97% similarity after removal of the chimeric sequences using UPARSE (version 7.1 https://drive5.com/uparse/). The phylogenetic affiliation of each 16S rRNA gene sequence was analyzed by RDP classifier (https://rdp.cme.msu.edu/) against the RDP database (RDP Release 11) using a confidence threshold of 70%. Sample diversity metrics were assessed based on the nonparametric Shannon–Wiener (SW) diversity index and Simpson's diversity index. The nonparametric Mann–Whitney *U* test was used to test for significant differences between the two groups. Comparison between multiple groups was done using a nonparametric Kruskal–Wallis test. Raw sequencing data of the 16S rRNA gene V3-V4 regions and accompanying information are available in the Sequence Read Archive database under accession number PRJNA736765.

### 2.10. Statistical Analysis

The data were expressed as mean ± SD. SPSS 21.0 software was used for statistical analysis. One-way analysis of variance was used for comparison between groups. A *p*-value of <0.05 was considered statistically significant.

## 3. Results

### 3.1. Effect of HQ-HH on Cerebral Infarction and Neuroethology in Rats with Ischemic Stroke

**Figure 1:**
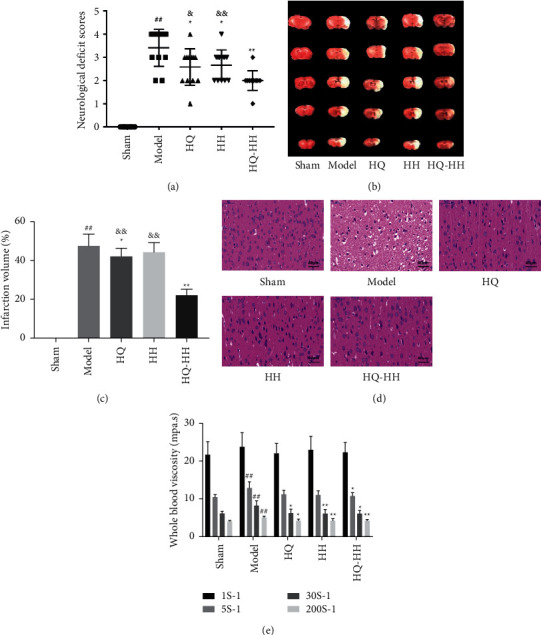
Effect of HQ-HH treatment on cerebral I/R injury in rats. HQ-HH treatment significantly improved the neurological scores and infarct volumes, decreased neuronal damage, and reduced whole blood viscosity at different shear rates compared with the MCAO group. (a) Neurological score. (b) Representative images of TTC staining. (c) Infarct volume. (d) Representative images of HE staining. (e) Comparison of the whole blood viscosity at different shear rates of rats in each group after 24h of MCAO/R. ##*P* < 0.01 versus sham; ^*∗*^*P* < 0.05, ^*∗∗*^*P* < 0.01 versus model; and ^&^*P* < 0.05, ^&&^*P* < 0.01 versus HQ-HH. Mean ± SD (*n* = 10 for neurological score, *n* = 12 for infarct volume and brain oedema, and *n* = 6 per group for the whole blood viscosity at different shear rates).

In this study, we evaluated the protective effects of HQ-HH against MCAO in rats. Compared with the sham group, the model group had significantly damaged neurological function (*P* < 0.01), suggesting that the MCAO with QDBS model was successfully established (Figure 1(a)). Compared with HQ and HH groups, the HQ-HH group had a significantly improved neurological function score (*P* < 0.05 and *P* < 0.01), demonstrating that HQ-HH significantly improves the neurological function of rats with CI/RI. The infarct volume of rats in the HQ-HH group was significantly reduced as compared with that in the model group (*P* < 0.05 and *P* < 0.01) (Figures 1(b) and 1(c)). Compared with the HQ and HH group rats, HQ-HH group rats had significantly reduced volume of cerebral infarction (*P* < 0.01), suggesting that HQ-HH reduces the volume of cerebral infarction in rats with CI/RI. Among HH, HQ, and HQ-HH, HQ-HH has the best protective effect against CI/RI.

### 3.2. HQ-HH Ameliorates MCAO-Induced Neuronal Injury

To explore the neuroprotective effect of HQ-HH on neuronal injury caused by MCAO, we stained the nerve cells of rats of different groups with HE and observed the morphological changes (Figure 1(d)). Compared with the normal morphology of nerve cells in the sham group, the morphology of the nerve cells in the model group was significantly changed. Most of the cells were disordered with a pyknotic or severely atrophied nucleus. Compared with the model group, the HQ and HH groups had improved morphology of cerebral cortex cells, but some neurons still showed abnormal morphology. The intercellular space was significantly smaller, and the morphology was distinguishable in the HQ-HH group than the model group. Furthermore, in the HQ-HH group, the dead neurons were less in number. Therefore, HQ-HH has visible protective effects on nerve cells, and the protective effect of HQ-HH is better than the protective effects of HQ and HH.

### 3.3. Whole Blood Viscosity Evaluation at Different Shear Rates

Compared with the sham group rats, model group rats had significantly increased whole blood viscosities at three shear rates: 5s-^1^, 30s^−1^, and 200s^−1^(*P* < 0.01). These findings indicate that CI/RI caused by thrombus removal increases the whole blood viscosity in rats (Figure 1(e)). The whole blood viscosity in the HQ-HH group was significantly reduced as compared with that in the model group; the reduction in viscosity in the HQ-HH group was significantly greater than the corresponding reductions in HH and HQ groups (*P* < 0.05 and *P* < 0.01).

### 3.4. Role of Autophagy in I/R and Beneficial Effects of HQ-HH

**Figure 2:**
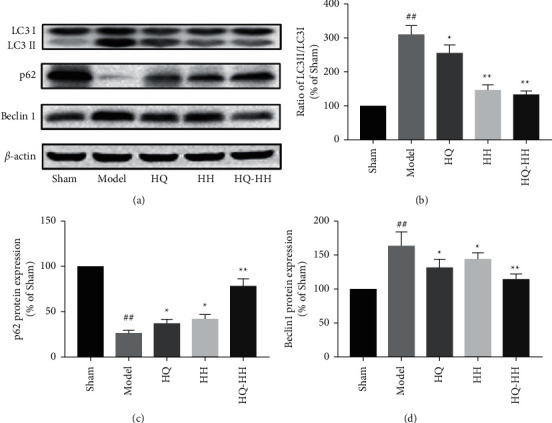
The expression level of autophagy marker protein in each group after 24 hours of MCAO/R. (a) The expression of LC3, p62, Beclin1 as shown in Western blot. (b) The ratio of LC3II/LC3I was significantly decreased after HQ-HH administration. (c) The protein expression of p62 was significantly increased after HQ-HH administration. (d) The protein expression of Beclin1 was significantly decreased after HQ-HH administration. Mean ± SD. ^##^*P* < 0.01 versus sham; ^*∗*^*P* < 0.05, ^*∗∗*^*P* < 0.01 versus model; ^&&^*P* < 0.01 versus HQ-HH. *n* = 3 per group.

**Figure 3:**
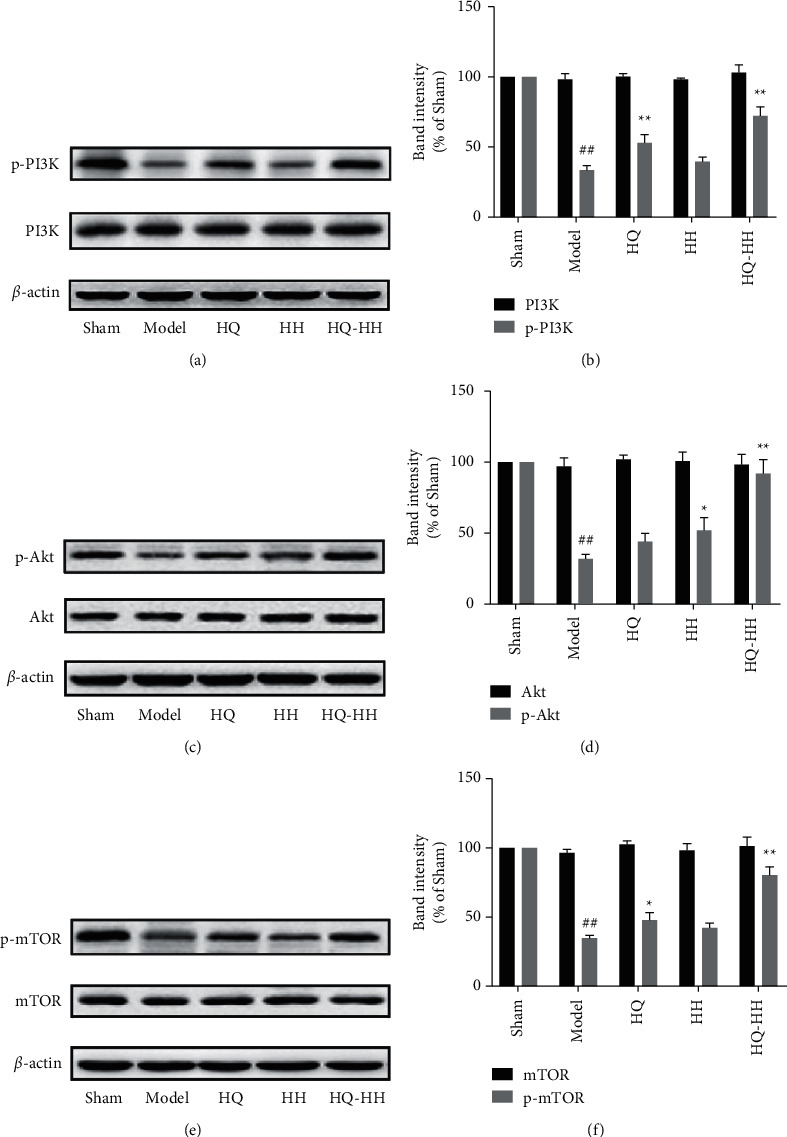
The effect of the expression level of PI3K-Akt-mTOR signaling pathway in each group after 24 hours of MCAO/R. (a) The expression of PI3K and p-PI3K as shown in Western blot. (b) The band intensity of p-PI3K was significantly increased after HQ-HH administration. (c) The expression of Akt and p-Akt as shown in Western blot. (d) The band intensity of p-Akt was significantly increased after HQ-HH administration. (e) The expression of mTOR and p-mTOR as shown in Western blot. (f) The band intensity of p-mTOR was significantly increased after HQ-HH administration. Mean ± SD, *n* = 3 per group.

As compared with the model group, the HQ-HH group had significantly lower (*P* < 0.01) levels of autophagy marker proteins LC3II/LC3I and Beclin1 and significantly higher (*P* < 0.01) p62 levels, 24 hours after I/R induction. These findings highlight the changes of autophagy. Compared with the HQ and HH groups, the HQ-HH group had significantly reduced levels of LC3II/LC3I and significantly increased levels of p62 (*P* < 0.01, Figure 2). No significant differences (*P* > 0.05) were observed in the levels of PI3K-Akt-mTOR signaling pathway proteins (PI3K, Akt, and mTOR) between model and sham groups, but the phosphorylation levels of PI3K, Akt, and mTOR were significantly reduced in the model group (*P* < 0.01). The HQ-HH group had significantly higher (*P* < 0.01) PI3K, Akt, and mTOR phosphorylation levels as compared with the model, HQ, and HH groups. These findings suggest that the combined effect of HQ and HH is superior to the effect of HQ and HH (Figure 3).

### 3.5. HQ-HH Altered the Composition and Function of Gut Microbiota

**Figure 4:**
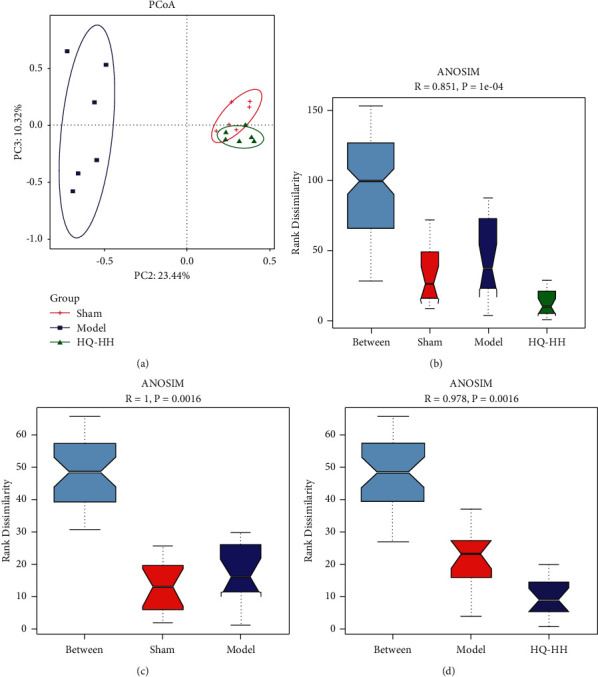
HQ-HH altered the composition and function of gut microbiota. (a) Principal coordinate analysis (PCoA) of weighted UniFrac distance shows that sham, model, and HQ-HH groups have obvious separation in microbial composition. (b–d) ANOSIM box plot analysis.

**Figure 5:**
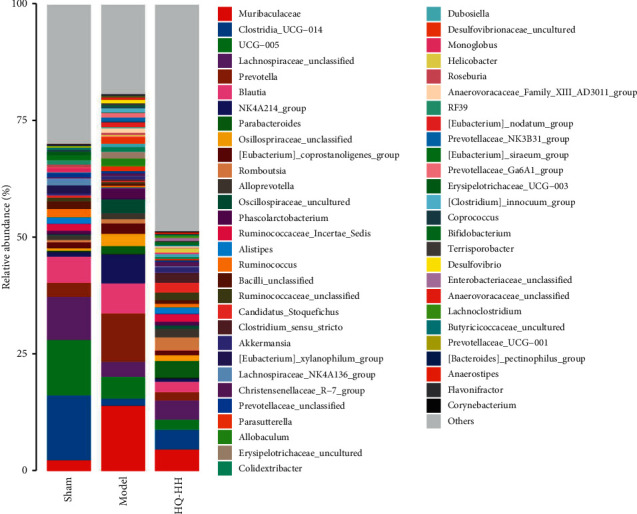
Comparison of bacterial community structure and relative abundance.

Based on the abundance distribution of OTUs in samples, we analyzed the *β*-diversity of the intestinal bacterial community and observed the differences in community compositions of different groups. The principal coordinate analysis (PCoA) results based on Bray–Curtis distance are shown in Figure 4(a). The distance between the sample points of the model group and the sham group was high, indicating that the bacterial composition of the model group is quite different from that of the sham group. The PCoA map of the HQ-HH group was similar to that of the sham group. The distance between the HQ-HH group and the model group was high, indicating that the composition of the HQ-HH group was more similar to the sham group as compared with the model group. The ANOSIM findings revealed that there were differences in the flora among the sham, model, and HQ-HH groups (Figure 4(b)). ANOSIM was used to further analyze the groups of two (Figures 4(c) and 4(d)). The differences between the sham group and the model group, as well as between the model group and the HQ-HH group, were significantly greater than those within the group. These findings indicate that there were significant differences in the flora composition between the sham group and the model group and between the model group and the HQ-HH group. To further explore the effects of HQ-HH on the taxonomy of intestinal flora in MCAO rats, we analyzed the significant floral changes in sham, model, and HQ-HH groups by variance analysis according to the species annotation and abundance information of fecal samples at the genus level. Compared with the model group, the HQ-HH group had a different relative abundance of *Lachnospiraceae_unclassified*, *Alistipes*, *Ruminococcus*, *Monoglobus*, *Lachnoclostridium*, *Anaerostipes*, *Muribaculaceae*, *Prevotella*, *Blautia*, *Phascolarctobacterium*, *Parasutterella, Colidextribacter,* and *Terrisporobacter* (Figure 5). These findings suggest that HQ-HH may affect the function of intestinal flora by changing the species structure and abundance of intestinal flora in MCAO rats.

## 4. Discussion

The incidence of ischemic stroke is increasing year by year. Clinical methods such as mechanical recanalization and recombinant tissue plasminogen activator can restore the cerebral blood flow, but the subsequent CI/RI is another obstacle that affects the prognosis [21]. Therefore, the development of effective anticerebral ischemia drugs with a clear mechanism of action is important [22]. TCM therapies have been historically used to prevent and treat ischemic stroke and have several advantages. TCM plants can control the disease by acting on many different targets. The theory of TCM believes that ischemic stroke belongs to the category of QDBS, in which Qi deficiency is the core pathogenesis and blood stasis is the fundamental pathogenesis [23]. Therefore, tonifying Qi, promoting blood circulation, and removing blood stasis by TCM dialectical treatment are beneficial. HQ is a traditional medicine for invigorating Qi. As per the Chinese Pharmacopoeia, HQ is often used for the treatment of “Qi deficiency” syndrome. HH is widely used in the treatment of cardiovascular and cerebrovascular diseases. It promotes blood circulation and removes blood stasis. HQ [24] and HH [25] have been found safe and effective in the treatment of CI/RI. Our previous study found that the combination of HQ and HH minimizes the brain damage caused by cerebral ischemia in rats and that the mechanism of brain protection is related to the antioxidant activity of this combination [26]. Therefore, investigating the role of autophagy in the process of cerebral ischemia treatment with HQ-HH is the main focus of the present study.

In this study, the effects of HQ-HH on cerebral infarction volume of MCAO rats were observed by TTC and HE staining. HQ-HH significantly improved the neuronal damage induced by MCAO. HQ-HH treatment also significantly altered neurological deficits. These findings validated that HQ-HH possesses a neuroprotective effect against MCAO-induced CI/RI. By comparing the whole blood viscosity of rats in each group 24h after cerebral I/R induction, we observed a decrease in the whole blood viscosity of HQ-HH group rats at four shear rates, indicating that HQ-HH improves the blood stasis state of brain I/R to a certain degree. These manifestations can reduce blood viscosity and improve brain microcirculation. Studies have also shown that autophagy plays a critical role in CI/RI, so the brain-protective effect of HQ-HH is apparent. Whether this effect is related to autophagy is the main focus of this study. This study aims to explore the neuroprotective mechanism of HQ-HH against brain I/R, especially the autophagy pathway.

Oxidative stress, excitotoxicity, autophagy, and apoptosis are involved in the development of CI/RI [27]. Oxidative stress is one of the earliest responses after an acute cerebral ischemic injury [28]. ROS can regulate the survival of damaged cells by affecting autophagy and apoptosis [29]. Therefore, to prove whether HQ-HH has an antioxidant effect, we evaluated whether HQ-HH inhibits autophagy in the treatment of cerebral ischemia. We used Western blot to determine the expression levels of autophagy marker proteins Beclin1, LC3, and p62 and evaluate the degree of autophagy after cerebral I/R. Beclin1 acts as an autophagy marker and inhibits autophagy [30]. LC3 protein includes LC3-I and LC3-II. During autophagy, LC3-I combines with lipid phosphatidylethanolamine (PE) to form LC3-II. The content of LC3-II positively correlates with the number of autophagosomes, partially reflecting the current autophagy activity of cells [31, 32]. p62 is involved in a variety of signal transduction processes. p62 binds to ubiquitinated proteins and forms a complex with LC3-II, which is then degraded in lysosomes; p62 is continuously consumed during autophagy [33]. We found a significant increase in p62 protein expression and a significant decrease in the levels of Beclin1 and LC3II/LC3I proteins after treatment with HQ-HH. We can conclude that HQ-HH inhibits the autophagy activity, improves cell homeostasis, and effectively improves the quality of life of MCAO rats and the symptoms of CI/RI.

As one of the classic autophagy signaling pathways, PI3K-Akt-mTOR is mainly involved in regulating migration, survival, and proliferation of the cells. Activation of the PI3K/Akt/mTOR pathway inhibits the occurrence of autophagy, which is essential for the growth and survival of neurons after cerebral ischemia [34, 35]. PI3K is an intracellular phosphatidylinositol kinase, and induction of autophagy depends on PI3K and Beclin1 [36]. Akt is the main downstream effector of PI3K. Activation of Akt can inhibit neuronal autophagy and promote neuronal proliferation and survival. mTOR is the key regulator of cell growth and metabolism and the downstream element of the PI3K-Akt signaling pathway. The increase of mTOR activity inhibits autophagy and restores the integrity of intracellular lysosomes, which play a vital negative regulatory role in autophagy [37]. Exploring the drugs that activate the PI3K-Akt-mTOR signaling pathway to inhibit autophagy has guiding significance for clinical treatment of CI/RI. Our previous network pharmacology studies have shown that the PI3K-Akt signaling pathway may be involved in the synergistic effect of HQ and HH in the treatment of cerebral I/R [26]. In this study, we found that HQ-HH upregulates the levels of p-PI3K, p-Akt, and p-mTOR in MCAO rats with QDBS. This process activates the PI3K-Akt-mTOR signaling pathway and protects the brain tissue injury after I/R by inhibiting autophagy.

In recent years, the interactions between intestinal flora and diseases have received considerable attention from the scientific community [38]. The concept of the brain-gut-axis provides a new approach for the study of diseases [39]. Gut dysbacteriosis can lead to significant changes in the physiological processes and cause severe intestinal inflammation, imbalances in intestinal redox status, and autophagy [40]. In these processes, autophagy, as an innate barrier of infection [41], plays a vital role in identifying and destroying intracellular pathogens [42]. Loss of the intestinal barrier leads to systemic immune activation and extensive extraintestinal autoimmune and inflammatory diseases [43]. These processes are affected by the composition and function of intestinal flora [44]. Studies have shown that the relationship between autophagy and microorganisms determines the results of their interaction [41]. The exogenous fecal microflora increases FoxO-mediated autophagy in receptor intestinal mucosa [45]. Consistent with these results, our findings validated the relationships between autophagy and intestinal flora. The composition and structure of intestinal microflora in the model group were different from those in the sham group; however, administration of HQ-HH prevented the imbalance in the original microflora and the microflora was found similar to that of the sham group. Integrating the intestinal microflora data was helpful in identifying the effective intestinal microflora involved in the treatment of ischemic stroke by HQ-HH and provided new treatment ideas. Compared with the model group, the HQ-HH group had different relative abundance of *Lachnospiraceae_unclassified*, *Alistipes*, *Ruminococcus*, *Monoglobus*, *Lachnoclostridium*, *Anaerostipes*, *Muribaculaceae*, *Prevotella*, *Blautia*, *Phascolarctobacterium*, *Parasutterella*, *Colidextribacter*, and *Terrisporobacter*. In the MCAO rats, the relative abundance of intestinal *Lachnospiraceae_unclassified* and *Anaerostipes* was significantly higher while that of *Blautia* and *Phascolarctobacterium* was significantly lower as compared to the sham group. Huws et al. [46] found that *Lachnospiraceae_unclassified* may play a predominant role in ruminal biohydrogenation. Bui et al. [47] demonstrated that *Anaerostipes rhamnosivorans* promotes the formation of butyric acid and may benefit colon health. On the contrary, *Blautia* was the only gut microbe found to be significantly and inversely associated with visceral fat, regardless of sex [48]. Fan et al. [49] found that the relative abundance of *Phascolarctobacterium* in the intestinal flora of patients with poststroke depression was higher than that of the healthy patients. In conclusion, HQ-HH can improve the intestinal flora imbalance after ischemic stroke and promotes the recovery of healthy intestinal flora. We demonstrated that the efficacy of HQ-HH is higher than that of HH or HQ alone. Further, we used PCoA to analyze the intestinal flora of rats. The difference between the intestinal flora of the model group and the sham group was relatively large, and administration of HQ-HH prevented the deviation from the original composition of intestinal flora. The ANOSIM analysis results further strengthened our hypothesis. Taken together, we demonstrated that CI/RI leads to floral imbalance, and administration of HQ-HH can prevent the fluctuations in the original flora. The effect of HQ-HH on intestinal flora may be an important mechanism underlying its ability to treat ischemic stroke.

Autophagy is a process of self-protection when the body is stimulated by adverse factors of internal and external environment. In fact, after a stroke, the body's immune function is significantly reduced, and autophagic response and intestinal microbiome disorders occur. At the same time, the intestinal floral imbalance affects the immune response, severity, stroke outcome, and related poststroke complications [50]. Recently, emerging research has focused on the connection between inflammation and autophagy [51]. Accumulating evidence indicates that inflammation and autophagy are linked by reciprocal regulation, and they are orchestrated by the upstream mTOR pathway [52]. We confirmed that HQ-HH can treat ischemic stroke through the PI3K-Akt-mTOR autophagy pathway and showed that HQ-HH can promote the recovery of intestinal flora in rats after ischemic stroke. However, the relationship between autophagy and intestinal flora needs to be further studied. We hypothesized that stroke changes intestinal microbiota and that the metabolites derived from intestinal microbiota, such as short-chain fatty acids, bile acids, and amino acids, may act as chemical messengers mediating the host-microbe interactions. In the future, we plan to further validate this mechanism by collecting samples at different time points and perform cell culture and fecal microbiota transplantation studies. In short, a comprehensive understanding of the changes of intestinal flora during the onset of ischemic stroke and the relationship between intestinal flora and brain is expected to achieve breakthrough results in the prevention and treatment of central nervous system diseases.

## 5. Conclusion

Does autophagy have more advantages than disadvantages or more disadvantages than advantages? However, there is no doubt that autophagy is indeed involved in the pathophysiology of ischemic stroke. More and more evidence also shows that autophagy is a regulated self-digestion process, which is of great significance to maintaining intestinal homeostasis.

In conclusion, we demonstrated that HQ-HH significantly protects against brain I/R injury in MCAO rats with QDBS by activating the PI3K-Akt-mTOR signaling pathway and inhibiting autophagy. In addition, HQ-HH prevents imbalance in intestinal flora after ischemic stroke. HQ-HH may treat CI/RI by preventing the changes in the intestinal microbial structure and inducing inhibit autophagy. These findings provide a new theoretical basis for the protective mechanism of HQ-HH in the treatment of cerebral I/R injury.

## Data Availability

The data used to support the findings of this study are included in the article.

## Abbreviations

CI/RI: Cerebral ischemia/reperfusion injury
HQ-HH: Huangqi-Honghua combination
HQ: Huangqi
HH: Honghua
TCM: Traditional Chinese medicine
QDBS: Qi deficiency and blood stasis
MCAO: Middle cerebral artery occlusion
TTC: 2,3,5-Triphenyl tetrazolium chloride
HE: Hematoxylin-eosin
Akt: Protein kinase B
mTOR: Mammalian target of rapamycin
PI3K: Phosphatidylinositol 3-kinase
ANOSIM: Analysis of Similarities
MCAO/R: Middle cerebral artery occlusion/reperfusion
ROS: Reactive oxygen species.
